# “You Have to Love It”

**DOI:** 10.31486/toj.24.5040

**Published:** 2024

**Authors:** Ronald G. Amedee

**Affiliations:** Director of Clinical School and Professor, The University of Queensland Medical School, Ochsner Clinical School, New Orleans, LA; Editor-in-Chief, *Ochsner Journal*

*Whatever you want to do, if you want to be great at it, you have to love it and be able to make sacrifices for it.*

*–Maya Angelou*

One of the standout features of the Spring 2024 issue of *Ochsner Journal* is an absolute gut-punch of a Health, Medicine, and Society column about a free walk-in clinic at the Ozanam Inn in New Orleans where people experiencing homelessness are welcomed without judgment and are provided with care. In a profoundly moving call to action, Elyse Stevens of LSU Internal Medicine yanks back the curtain that separates *us* from *them* and provides a thought-provoking perspective on the myriad obstacles this stigmatized population faces in seeking and obtaining medical treatment.

In addition, four original research articles, two literature reviews, and seven case reports and clinical observations serve as the principal elements for this edition, with the majority of the articles in the issue coauthored by Ochsner staff.

All four original research articles have Ochsner physician authors. Ochsner Orthopedics/Sports Medicine continues its strong support of the *Journal* with an important study—“Comparative, Controlled, Retrospective Study of Patient-Reported Outcomes After Meniscectomy With Adjunctive Use of Platelet-Rich Plasma or Amniotic Umbilical Cord Tissue”—by Duru, Williams, Assid, Renshaw, and Jones. Ochsner Plastic and Reconstructive Surgery contributed “Outcomes of Reduction Mammaplasty in Adolescents vs Average-Age Patients: A 3-Year Single-Center Retrospective Analysis,” authored by Hajebian, Puyana, Babycos, and Friel. The Ochsner-Xavier Institute for Health Equity and Research and the Department of Obstetrics and Gynecology collaborated on “Evaluating Racial Disparities in Implementation and Monitoring of a Remote Blood Pressure Program in a Pregnant Population—A Retrospective Cohort Study” by Howard, Gillispie-Bell, Olet, Glenn, Ammar, and Price-Haywood. Ochsner ophthalmologist H. Sprague Eustis Jr. is senior author on “Methadone for Emergence Delirium in Ambulatory Pediatric Strabismus Surgery.”

Scullen, Milburn, and Mathkour, Ochsner physicians in Neurosurgery and Radiology, contributed to “Training Cerebrovascular and Neuroendovascular Surgery Residents: A Systematic Literature Review and Recommendations.” Another Neurology contribution, with assistance from Tulane and Ochsner's Department of Sports Medicine, is the case report, “Carotid Web as a Source of Thromboembolism in a Young African American Female.” Yet another contribution from Ochsner Orthopedics/Sports Medicine is the case report “Novel Allograft in the Load-Bearing Portion of the Femoral Head,” by Renshaw, Duru, Assid, Williams, Suri, and Jones. Ochsner physical therapist Jordan Hill provided “Evidence for Combining Conservative Treatments for Adhesive Capsulitis.”

As we come to the end of February 2024 with a leap year 29th day included, I am thinking back to just a month ago when I was in Brisbane, Australia, to onboard our new cohort of year-1 students entering The University of Queensland Medical School-Ochsner Clinical School program. As these students were about to commence their multiyear journey to become competent and caring physicians, what could I say to them to encourage and guide their future paths? Pondering this question, I looked back to a day more than four decades ago when I sat in medical school orientation just as they were now, and the above quote from Maya Angelou came to mind. To me, the quote best sums up our life's work in medicine. You must love it and be willing to personally make sacrifices for it. This sentiment is true for most occupations but should always resonate even more clearly in the mind and heart of each health care provider.

